# Lysophosphatidic acid enhances survival of human CD34^+^ cells in ischemic conditions

**DOI:** 10.1038/srep16406

**Published:** 2015-11-10

**Authors:** Ivana Kostic, Isabel Fidalgo-Carvalho, Sezin Aday, Helena Vazão, Tiago Carvalheiro, Mário Grãos, António Duarte, Carla Cardoso, Lino Gonçalves, Lina Carvalho, Artur Paiva, Lino Ferreira

**Affiliations:** 1 Biocant, Cantanhede, Portugal; 2 CNC-Center for Neuroscience and Cell Biology, University of Coimbra, Coimbra, Portugal; 3 Portuguese Institute for Blood and Transplantation, IP, Coimbra, Portugal; 4 Department of Cardiology, Coimbra University Hospital Center & Faculty of Medicine University of Coimbra, Coimbra, Portugal; 5 Crioestaminal, Cantanhede, Portugal; 6 Department of Anatomical Pathology, University Hospital of Coimbra, Coimbra, Portugal

## Abstract

Several clinical trials are exploring therapeutic effect of human CD34^+^ cells in ischemic diseases, including myocardial infarction. Unfortunately, most of the cells die few days after delivery. Herein we show that lysophosphatidic acid (LPA)-treated human umbilical cord blood-derived CD34^+^ cells cultured under hypoxic and serum-deprived conditions present 2.2-fold and 1.3-fold higher survival relatively to non-treated cells and prostaglandin E_2_-treated cells, respectively. The pro-survival effect of LPA is concentration- and time-dependent and it is mediated by the activation of peroxisome proliferator-activator receptor γ (PPARγ) and downstream, by the activation of pro-survival ERK and Akt signaling pathways and the inhibition of mitochondrial apoptotic pathway. In hypoxia and serum-deprived culture conditions, LPA induces CD34^+^ cell proliferation without maintaining the their undifferentiating state, and enhances IL-8, IL-6 and G-CSF secretion during the first 12 h compared to non-treated cells. LPA-treated CD34^+^ cells delivered in fibrin gels have enhanced survival and improved cardiac fractional shortening at 2 weeks on rat infarcted hearts as compared to hearts treated with placebo. We have developed a new platform to enhance the survival of CD34^+^ cells using a natural and cost-effective ligand and demonstrated its utility in the preservation of the functionality of the heart after infarction.

Cardiovascular diseases are responsible for the deaths of more than 4 million people in Europe every year. About 20 percent of these deaths are related to ischemic heart disease. Although endogenous stem cells are mobilized from the bone marrow during ischemic episodes, endogenous resources may not provide a critical mass capable of rescuing tissue from ischemic injury^1^. Therefore, the use of exogenous stem cells as a potential therapeutic approach to treat ischemic diseases is under evaluation. CD34^+^ cells represent an effective angiogenic stem cell component and early-phase clinical trials have shown that intramyocardial administration of autologous CD34^+^ cells may improve the functional capacity and symptoms of angina and chronic myocardial ischemia^2^,^3^. In addition, several pre-clinical studies have shown that CD34^+^ cells transplanted into the infarcted myocardium promote angiogenesis and preserve its functionality^4^,^5^. For therapeutic efficacy, it is imperative that stem cells or their progenies survive and engraft into the host tissue. Unfortunately, most of the cells die a few days after delivery and thus compromise the final outcome of the procedure^6^.

One of the first stresses that the cells encounter during the engraftment process is ischemia^7^. Injected cells tend to form clumps that are forced into potential interstitial spaces between tissue elements. Even in the context of well-vascularized tissue, these clumps are avascular, so diffusion is the only source of nutrient and oxygen transport until angiogenesis provides a vasculature. Some methodologies have been proposed to augment cell survival in ischemic conditions including the exposure of donor cells to temperature shock, genetic modification to overexpress growth factors, transduction of anti-apoptotic proteins, co-transplant of cells, or preconditioning the cells with pharmacological agents and cytokines (reviewed in refs 7,8). Despite these advances, the proposed methodologies have shown limited effectiveness due to the multi-factorial nature of cell death^7^, some of them are not cost-effective (for example the ones involving recombinant proteins) or are difficult to implement from a regulatory stand-point (for example genetic manipulation of the cells^4^, co-transplant of cells that are processed in the laboratory^9^).

Here we investigated the pro-survival activity of lysophosphatidic acid (LPA) in CD34^+^ cells. We have used umbilical cord blood CD34^+^ cells because we had easy access to cord blood samples and because previous studies have demonstrated the regenerative potential of these cells in the setting of myocardial infarction^6^,^10^,^11^. LPA is a natural phospholipid present in blood serum in micromolar ranges^12^. It increases at least two fold in the serum of patients after an acute myocardial infarction^13^. Studies have shown that LPA prevents apoptosis in hypoxic and serum-deprived mesenchymal stem cells^14^, serum-deprived fibroblasts^15^, Schwann cells^16^, renal tubular cells^17^, macrophages^18^, and hypoxia-challenged neonatal cardiomyocytes^19^. So far, little is know about the role of LPA in human hematopoietic stem/progenitor cells. Recent studies have examined the role of LPA in the differentiation of CD34^+^ cells^20^,^21^ but not in CD34^+^ survival under ischemic conditions. We hypothesize that LPA enhances the survival of CD34^+^ cells in ischemic conditions. To verify this hypothesis, we have evaluated the survival of human CD34^+^ cells in suspension or encapsulated in fibrin gels under hypoxia and serum-deprivation conditions. We have studied the survival mechanism using pharmacological inhibitors, LPA receptor expression and activation of pro-survival/inhibition of pro-apoptotic signaling pathways. We have further evaluated the proliferation, differentiation and secretome of LPA-treated versus non-treated CD34^+^ cells. Finally, we have evaluated *in vivo* CD34^+^ cell survival and its therapeutic effect in the preservation of cardiac function.

## Results

### LPA induces CD34^+^ cell survival in hypoxia and serum-deprivation conditions

**Figure 1:**
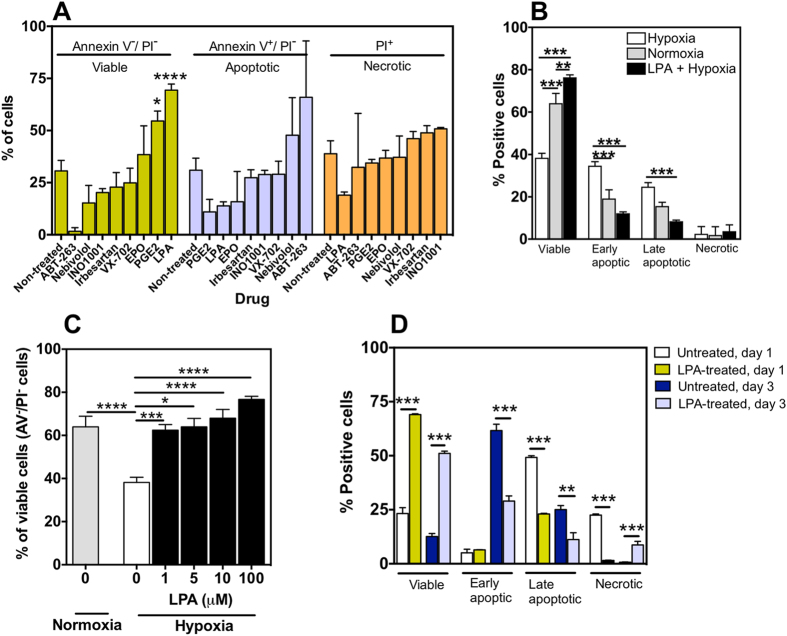
Assessment of CD34^+^ cell survival under hypoxia and serum-deprivation culture conditions: effect of LPA treatment and cell encapsulation. In all studies, CD34^+^ cells were isolated immediately from cord blood, without cryopreservation. (**A**) Survival, apoptosis and necrosis of CD34^+^ cells cultured in hypoxia for 24 h in serum-free medium with or without drugs. Studies were performed in CD34^+^ cells from 3–6 different donors (with the exception of ABT-263, where 2 donors have been used); 1–8 technical replicates per donor. (**B**) Survival, apoptosis and necrosis of CD34^+^ cells cultured in serum-free medium for 24 h in normoxia, hypoxia and hypoxia with LPA treatment. Studies performed in CD34^+^ cells from 4 different donors; 1–8 technical replicates per donor. (**C**) Effect of LPA concentration on the survival of CD34^+^ cells cultured in serum-free medium for 24 h in hypoxia (black and white columns) or normoxia (grey column). Studies performed in CD34^+^ cells from 3–12 different donors; 1–6 technical replicates per donor. (**D**) Non-treated or LPA-treated cells (1 × 10^6^ cells) encapsulated in fibrin gels (200 μL) were cultured in serum free medium with or without LPA (100 μM) under hypoxia conditions for 1 or 3 days. Studies performed in CD34^+^ cells from 3-different donors; 1 technical replicate per donor. *Denotes statistical significance: *P < 0.05, **P < 0.01, ***P < 0.001, ****P < 0.0001. In (**A**,**C**–**E**) results are presented as mean ± SEM.

Human umbilical cord blood-derived CD34^+^ cells (2 × 10^5^ cells per well of a 96-well plate) were incubated in X-Vivo medium (previously used in clinical trials^22^) under hypoxic conditions (0.5% O_2_) at 37 °C, for 24 h. The pro-survival effect of LPA as well as drugs approved by FDA for the treatment of cardiovascular diseases (e.g. Nebivolol^23^, Irbesartan^24^) and drugs being evaluated in pre-clinical/clinical assays to improve heart function in patients/models with heart failure (e.g. INO1001^25^, erythropoietin (EPO)^26^, VX-702^27^) was evaluated (Fig. 1A,B). For comparison purposes, we have tested the drugs at a concentration of 10 μM. This concentration has no cytotoxic effect (results now shown) and is slightly higher than the screenings performed before for CD34^+^ cell expansion^28^. Drug treatment was maintained during the duration of the assay. ABT-263, a pro-apoptotic molecule, was used as positive control for apoptosis. According to flow cytometry results, non-treated CD34^+^ cells show a very poor survival, with only ~31% viable cells after 24 h (Fig. 1A). Among seven drugs tested, LPA (*P* < 0.0001) and prostaglandin E2 (PGE2; *P* < 0.05) improved significantly cell survival (Fig. 1A). LPA was the drug with the highest pro-survival effect (~69% viable cells) and therefore further studied. LPA improved cell survival by reverting necrosis and apoptosis of CD34^+^ cells under hypoxic and serum-deprivation conditions (Fig. 1B).

The pro-survival effect of LPA is concentration dependent (between 1 and 100 μM) being the survival of CD34^+^ cells already statistically significant (*P* < 0.001) as compared to the control (non-treated cells) at 1 μM LPA (Fig. 1C). Importantly, the percentage of viable cells in CD34^+^ cells treated with 1 μM LPA and cultured under hypoxia conditions is similar to non-treated cells cultured under normoxia conditions. For subsequent tests a concentration of 100 μM has been used since the survival effect was maximal. The pro-survival effect of LPA decreases as a function of hypoxia time (Supplementary Figure 1). The percentage of viable cells in LPA-treated CD34^+^ cells decreased from 78% at day 1 to 40% at day 3. All together, the results obtained indicate that LPA is a pro-survival molecule for CD34^+^ cells and its effect is time and concentration-dependent.

The pro-survival effect of LPA is observed in CD34^+^ cells encapsulated in fibrin gels. Injectable scaffolds are very promising vehicles to deliver stem cells for regenerative medicine since they provide a favorable structural support for cell survival and proliferation^9^,^29^,^30^. Several studies have shown that cells transplanted with scaffolds in the cardiac setting improved cell survival, induced angiogenesis and preserved cardiac function after infarction^30^,^31^,^32^,^33^. Therefore we examined whether LPA could have a similar pro-survival effect in cells encapsulated in fibrin gels and cultured under hypoxic conditions. Non-treated CD34^+^ cells had poor survival in fibrin gels (23.2 ± 2.8% viable cells, *n* = 3, at day 1; 12.6 ± 1.2% viable cells, *n* = 5, at day 3) showing that the matrix alone did not have any pro-survival effect (Fig. 1D). In contrast, LPA-treated CD34^+^ cells encapsulated in fibrin gels presented high survival at later times (day 3) relatively to LPA-treated cells not encapsulated in fibrin gels (day 1: 69.0 ± 0.7% *vs* 76.7 ± 1.5% for encapsulated and non-encapsulated, respectively; day 3: 51.1 ± 0.8% *vs* 37.2 ± 6.6% for encapsulated and non-encapsulated, respectively). Overall, the results indicate that the pro-survival effect of LPA is also observed in cells encapsulated in fibrin gels.

### LPA induces CD34^+^ cell survival mainly through peroxisome proliferator-activated receptor γ (PPAR-γ)

**Figure 2:**
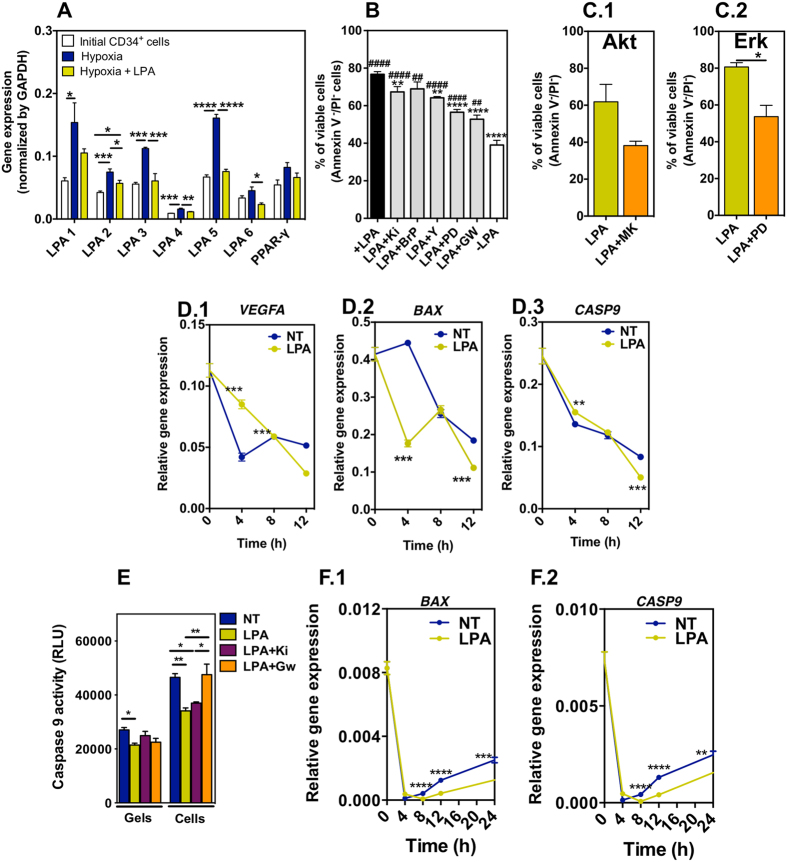
Survival mechanism in LPA-treated CD34^+^ cells. (**A**) Expression of LPA receptors. Cells were cultured in the different experimental conditions for 24 h. Gene expression was normalized by the expression of *GAPDH*.Studies were performed in CD34^+^ cells isolated from 3 different donors; 4 technical replicates. (**B**) Involvement of LPA receptors in cell survival. Cells were pre-treated with Rho kinase inhibitor (Y-2762), MEK1/2 inhibitor (PD98059), LPA_1_- and LPA_3_-specific inhibitor (Ki16425), a pan-G protein inhibitor (1-bromo-3(S)-hydroxy-4-(palmitoyloxy)butyl]phosphonate (BrP)), or peroxisome proliferator-activator receptor γ (PPAR-γ) inhibitor (GW9662) for 1 h before hypoxia and cell medium containing LPA (100 μM) for 24 h. Cells without any pre-treatment and cultured in serum-free medium with or without LPA, in hypoxia for 24 h, were used as positive and negative controls, respectively. Studies were performed in CD34^+^ cells isolated from at least 3 different donors; 1–8 technical replicates. # and * means statistical difference relatively to cells without any pre-treatment and cultured in serum free medium without LPA, and with LPA, respectively. (**C**) Percentage of viable cells was measured by FACS. CD34^+^ cells in hypoxia and serum deprived conditions were treated with LPA, LPA with MEK1/2 inhibitor (PD98059; an upstream activator of MAPK/Erk), or LPA with Akt inhibitor (MK2206). Studies were performed in CD34^+^ cells isolated from 3 different donors. (**D**) Gene expression of *VEGFA*, *BAX* and *CASP9*. NT and LPA mean non-treated and LPA-treated CD34^+^ cells in suspension. Gene expression was normalized by the expression of *GAPDH*.(**E**) Caspase 9 activity in LPA-treated or non-treated CD34^+^ cells either in suspension (Cells) or encapsulated in a fibrin gel (Gels) at 24 h. Activity was assessed by Caspase Glo® 9 Assay kit. (**F**) Gene expression of *BAX* and *CASP9* in fibrin gel encapsulated cells. Gene expression was normalized by the expression of *GAPDH*. In (**D**–**F**) studies were performed in CD34^+^ cells isolated from 3 different donors; 4 technical replicates. *^,#^Denotes statistical significance: *^,#^*P* < 0.05, **^,##^*P* < 0.01, ***^,###^*P* < 0.001, ****^,####^*P* < 0.0001. In all graphs, results are presented as mean ± SEM.

To date, up to six LPA receptors (LPARs) have been identified: LPA1-LPA6^34^. Studies also indicate that LPA has a high affinity for peroxisome proliferator-activator receptor γ (PPAR-γ)^35^,^36^. To identify the expression of all known LPA receptors, we performed real time polymerase chain reaction (qRT-PCR). CD34^+^ cells expressed LPARs 1–6 and PPAR-γ receptors (Fig. 2A). When CD34^+^ cells were cultured in hypoxia conditions for 24 h in the absence of LPA, the expression of the receptors was upregulated relatively to original CD34^+^ cells collected from cord blood. This upregulation was likely due to the differentiation of the cells (see below) as been noted for mesenchymal stem cells^37^. When CD34^+^ cells were cultured in hypoxia conditions in the presence of LPA, in general the expression of the receptors was lower than in non-treated cells.

To identify the receptor that is mediating the pro-survival effect of LPA (100 μM), we used Ki16425, a LPA_1_- and LPA_3_-specific antagonist, 1-bromo-3(S)-hydroxy-4-(palmitoyloxy)butyl]phosphonate (BrP), a pan-G protein inhibitor, and GW9662, an antagonist of peroxisome proliferator-activator receptor γ (PPAR-γ). We complemented this approach by inhibiting downstream targets of the receptors including Rho kinase by Y-2762/ROCK and the upstream kinases and activators of MAPK/Erk, i.e. MEK1/2 by PD98059. It is known that all LPARs couple with and activate G proteins, which in turn activate MAPK (LPA1, LPA2, LPA3 and LPA4) and Rho kinase (LPA1, LPA2, LPA4 and LPA5)^38^. Cells were treated for 1 h in X-Vivo medium containing a specific antagonist followed by culture under hypoxia conditions for 24 h in the presence of LPA. Cell survival was assessed by FACS using Annexin V/PI staining. Inhibitor concentrations used in this experiment (without LPA treatment) did not affect cell viability (data not shown). Our results indicated that PPAR-γ was the main mediator of the pro-survival effect of LPA under the conditions tested (Fig. 2B). The antagonist of PPAR-γ significantly decreased (*P* < 0.001) the number of viable cells induced by LPA from ~77% to ~53%; however, it did not block totally the pro-survival effect of LPA, since cells without LPA had a survival of 39% (*P* < 0.01). The inhibition of LPA1 and LPA3 by Ki16425, or the inhibition of G protein signaling by BrP, had a relatively small effect in the survival of CD34^+^ cells (Fig. 2B). The number of viable cells decreased from 77% (+LPA) to 67% (in case of Ki16425, *P* < 0.01). Importantly, the inhibition of MAPK signaling pathway suppressed the pro-survival effect of LPA at higher levels than the inhibition of Rho kinase signaling pathway. Because the two signaling pathways are downstream targets of different LPARs (see above), this might indicate different contributions of each LPAR in the survival of CD34^+^ cells.

Since ERK1/2 and PI3K/Akt signaling pathways are very important for cell survival^14^,^15^, we evaluated whether these pathways were activated in CD34^+^ cells exposed to LPA. Cells were treated for 1 h in X-Vivo medium containing Akt antagonist MK2206 or the upstream kinases and activators of MAPK/Erk antagonist PD98059 followed by the addition of LPA and culture of the cells under hypoxia conditions for 24 h. Both antagonists significantly decreased the number of viable cells induced by LPA from ~60% to ~39% (Akt; *P* = 0.07) and from ~80% up to ~57% (Erk; *P* < 0.05), respectively (Fig. 2C). We complemented these results by analyzing the expression of *VEGFA*, a pro-survival factor. Indeed, previous studies have shown that LPA increased VEGF secretion in mesenchymal stem cells^39^,^40^. Our gene expression results showed that LPA-treated CD34^+^ cells expressed significantly higher levels of *VEGFA* than non-treated cells for the first 8 h of cell culture (Fig. 2D.1); however, the levels of VEGF protein, measured by an ELISA kit at time 24 h, were very low (below the sensitivity limit of the kit, i.e., 16 pg/mL).

Taken together, our data confirmed the expression of receptors LPA1-LPA6 and PPAR-γ receptors in CD34^+^ cells and showed that LPA pro-survival effect in CD34^+^ cells cultured under hypoxic and serum-deprivation conditions is mainly mediated by the activation of PPAR-γ receptor. Although further studies need to be performed to show that the inhibition of PPARγ reduces ERK1/2 and PI3K/Akt signaling pathways, our current results with Akt and MAPK/Erk antagonists in LPA-activated CD34^+^ cells indicate that both pathways are involved as downstream signaling events of the PPAR-γ receptor activation.

### LPA inhibits apoptosis by inhibiting the expression of BAX and caspase 9

The pro-survival mechanism of LPA was further studied at gene and protein levels. We asked whether the anti-apoptotic role of LPA was mediated by the inhibition of mitochondrial-dependent apoptotic pathway. It is known that the translocation of BAX from the cytoplasm to the mitochondrial membrane plays an important role in the release of cytochome c to the cytoplasm, which leads to the activation of caspase 9 and the initiation of cell apoptosis^41^,^42^,^43^. Our results showed that LPA down-regulated significantly *BAX* and *CASP9* (caspase *9* gene) expression in CD34^+^ cells after 4 h (*BAX*) and 12 h (*BAX* and *CASP9*) as compared to non-treated cells (Fig. 2D.2). Similarly, LPA down-regulated *BAX* (8–24 h) and *CASP9* (8–24 h) gene expression in CD34^+^ cells encapsulated in fibrin gels (Fig. 2F.1). These analyses were extended to the activity of caspase 9. LPA significantly inhibited the activity of caspase 9 in CD34^+^ cells cultured in ischemic conditions and the inhibition was reversed by PPAR-γ inhibitor (GW9662) but not LPA 1/3 inhibitor (Ki16425) (Fig. 2E). Therefore our results are in line with previous results showing that LPA protects T cells and mesenchymal stem cells from apoptosis by suppression of BAX, i.e., by the inhibition of mitochondrial-dependent apoptotic pathway^14^,^42^. However, the anti-apoptotic role of LPA in CD34^+^ cells is essentially mediated by PPARγ while in mesenchymal stem cells is mediated by LPA receptors.

### LPA induces cell proliferation but it is unable to maintain the undifferentiated state of CD34^+^ cells

**Figure 3:**
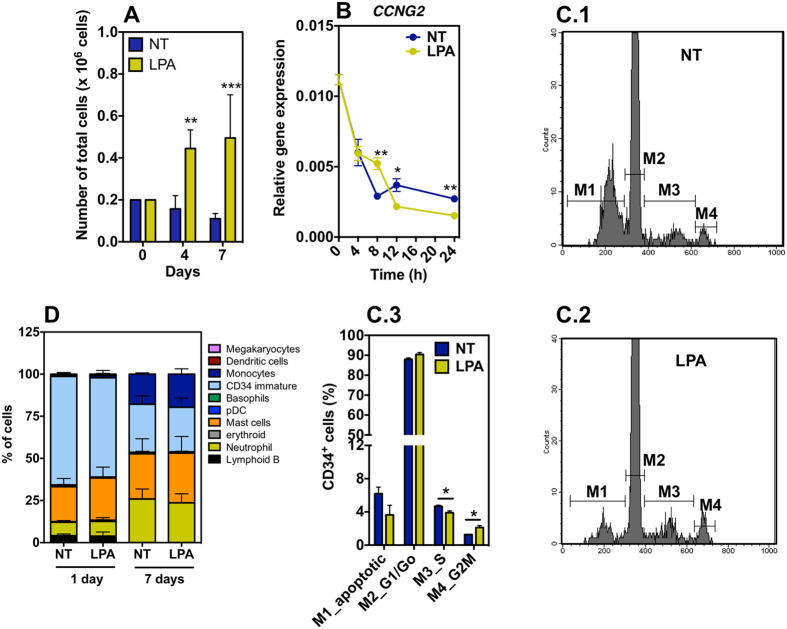
Proliferation and differentiation of LPA-treated CD34^+^ cells. (**A**) Number of non-treated and LPA-treated cells after 1 day in hypoxia and additional 3 (4^th^ day) or 6 days (7^th^ day) in normoxia. Cells were treated with 100 μM of LPA. (**B**) Gene expression of cyclin G (*CCNG2*) as assessed by qRT-PCR analyses. Gene expression was normalized by the expression of *GAPDH*.(**C**) CD34^+^ cell cycle, as evaluated by flow cytometry and PI staining, was performed in LPA- or non-treated cells. (**C.1**,**C.2**) show representative FACS results for each experimental group. (**D**) Cell differentiation as measured by flow cytometry using a cocktail of antibodies (please see Supplementary Table 2) to identify hematopoietic cell types. Cells were cultured in hypoxia for 1 day followed or not by 6 days in normoxia. In all graphs, cells were cultured with or without LPA (100 μM). In all studies were performed in CD34^+^ cells isolated from at least 3 different donors; 1–5 technical replicates. *Denotes statistical significance: *P < 0.05, **P < 0.01, ***P < 0.001. In all graphs, results are presented as mean ± SEM. In graphs (**A**–**C**) NT and LPA mean non-treated and LPA-treated CD34^+^ cells in suspension.

LPA is highly mitogenic in quiescent cells^44^. To determine whether LPA could induce the proliferation of CD34^+^ cells in hypoxia and serum-deprivation conditions, a suspension of non-treated or LPA-treated cells (2 × 10^5^ cells in 200 μL of X-vivo medium) was exposed to hypoxia for 24 h and then cultured under normoxia conditions for 6 additional days. LPA-treated cells increased their number approximately 3-fold over the 7 days period while non-treated cells decreased to half their initial number (Fig. 3A). Our results show that CD34^+^ cell proliferation is correlated with an up-regulation of cyclin G (CCNG2) at 8 h (Fig. 3B). We complemented these results by monitoring cell cycle by flow cytometry in cells cultured in the presence and absence of LPA for 24 h under hypoxic conditions. LPA-treated cells cycled more actively than the non-treated cells since more CD34^+^ cells were found in G2M phase (Fig. 3C). Because LPA-treated cells are in lower percentage in S-phase than non-treated cells it is likely that they are proliferating faster.

To examine the effect of LPA on CD34^+^ cell self-renewal/differentiation, CD34^+^ cells were cultured in X-vivo medium supplemented or not with LPA for 1 day under hypoxic conditions or cultured for 1 day in hypoxia followed by 6 days in normoxia. At the end, cells were characterized by flow cytometry for differentiation markers (Supplementary Tables 1 and 2, Supplementary Figures 2 and 3). After 1 day of hypoxia, both non-treated and LPA-treated CD34^+^ cells started to differentiate into mast cells (NT: 21.7 ± 9.8%, *n* = 4; LPA: 25.0 ± 13.0%, *n* = 4) and neutrophils (NT: 7.9 ± 2.0%, *n* = 4; LPA: 8.7 ± 4.4%, *n* = 4) (Fig. 3D). Only 64.3 ± 4.8% and 58.8 ± 8.7% of the non-treated or LPA-treated CD34^+^ cells, respectively, expressed CD34 marker. Cells cultured for 1 day in hypoxia and then 6 days in normoxia further differentiated into several cell lineages including dendritic cells (DCs), basophils, monocytes, among others. Similar differentiation profiles were observed for non-treated and LPA-treated cells, without statistical difference (*P* > 0.05). All together, our results show that LPA induces CD34^+^ cell proliferation but is unable to maintain their undifferentiated state.

### LPA modulates differently cytokine release in CD34^+^ cells and CD34^+^ cells encapsulated in fibrin gels

**Figure 4:**
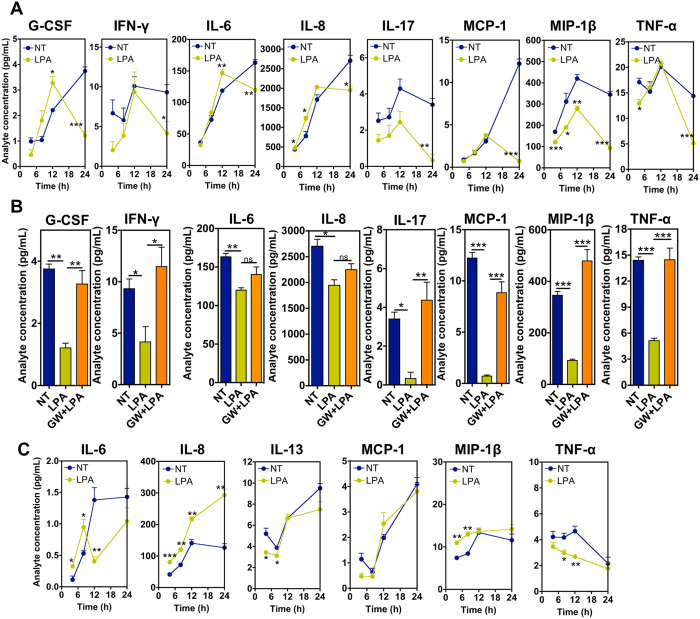
Secretome of LPA-treated CD34^+^ cells. (**A**) Cytokine secretion as assessed by Bio-Plex analyses for non-treated (NT) and LPA-treated (LPA) CD34^**+**^ cells (cells in suspension). (**B**) Cytokine secretion for non-treated (NT), LPA-treated (LPA) and LPA + PPAR-γ inhibitor (GW9662)-treated (GW + LPA) CD34^+^ cells at time point 24 h. Cells were in suspension. (**C**) Cytokine secretion for non-treated (NT) and LPA-treated (LPA) CD34^+^ cells encapsulated in fibrin gels. In (**A**–**C**) studies were performed in CD34^+^ cells isolated from 3 different donors; 4 technical replicates. *Denotes statistical significance: *P < 0.05, **P < 0.01, ***P < 0.001. In all graphs, results are presented as mean ± SEM.

The regenerative potential of CD34^+^ cells in ischemic diseases such as myocardial infarction is mainly mediated by the release of paracrine factors^45^. To determine the effect of LPA in the release of signaling cytokines by CD34^+^ cells and CD34^+^ cells entrapped in fibrin gels we used a cytokine bead array for the following cytokines: interleukin-1β (IL-1β), IL-2, IL-4, IL-5, IL-6, IL-7, IL-8; IL-10, IL-12(p70), IL-13, IL-17, granulocyte colony-stimulating factor (G-CSF), granulocyte/macrophage colony-stimulating factor (GM-CSF), interferon-γ (IFN-γ), monocyte chemotactic protein (monocyte chemotactic activating factor [MCP-1 (MCAF)], macrophage inflammatory protein-β (MIP-1β) and tumor necrosis factor-α (TNF-α). The cells were incubated in serum-free medium X-Vivo under hypoxia conditions and the samples were taken at different time points (4, 8, 12 and 24 h), either in the presence or absence of LPA (100 μM) and PPAR-γ-receptor antagonist GW9662. CD34^+^ cells in suspension (Fig. 4A,B) secreted much higher concentrations of cytokines than cells encapsulated in fibrin gels (Fig. 4C). The most secreted cytokine was IL-8 (cells only: 2704.4 ± 234.4 pg/mL; encapsulated cells: 126.4 ± 21.6 pg/mL, both at 24 h). During the first 12 h, cells in suspension secreted higher levels of IL-8, IL-6, and G-CSF than non-treated cells. During the same period of time, cells expressed lower levels of MIP-1β (times: 4, 8 and 12 h) and TNF-α (time: 4 h) than non-treated cells. At time 24 h, LPA-treated CD34^+^ cells secreted lower levels of all the measurable cytokines such as G-CSF, IFN-γ, IL-6, IL-8, IL-17, MCP-1, MIP-1β, and TNF-α than non-treated cells. Therefore, LPA decreased significantly the secretion of pro-inflammatory cytokines at 24 h. Importantly, the secretion of most cytokines (with the exception of IL-6 and IL-8) at 24 h was restored in cells inhibited with PPAR-γ-receptor antagonist GW9662, indicating that most of cytokine secretion was controlled by PPAR-γ receptor (Fig. 4B).

CD34^+^ cells encapsulated in fibrin gels (Fig. 4C) showed a different cytokine secretion profile than suspended CD34^+^ cells (Fig. 4A,B). For example, the secretion of IL-17, G-CSF and IFN-γ was not observed, while IL-13 cytokine was only observed in CD34^+^ cells encapsulated in fibrin gels. In fibrin gels, LPA-treated cells secreted higher levels of IL-6, IL-8 and MIP-1β than non-treated cells during the first 8 h. During the same time, cells expressed lower levels of IL-13 and TNF-α than non-treated cells. From 8 to 24 h, LPA-treated cells expressed lower levels of TNF-α but higher levels of IL-8 than non-treated cells.

Overall, after 24 h of treatment, LPA decreased the secretion of most of the anti-inflammatory cytokines in suspended CD34^+^ cells relatively to non-treated cells. With the exception of IL-6 and IL-8, the differences in cytokine secretion between non-treated and LPA-treated cells were abolished when the PPAR-γ inhibitor was used. Our results further show that the encapsulation of the cells in fibrin gels changed the magnitude and profile of cytokines secreted by non-treated and LPA-treated CD34^+^ cells.

### LPA-treated CD34^+^ cells entrapped in fibrin gels have higher survival *in vivo* after myocardial infarction

**Figure 5:**
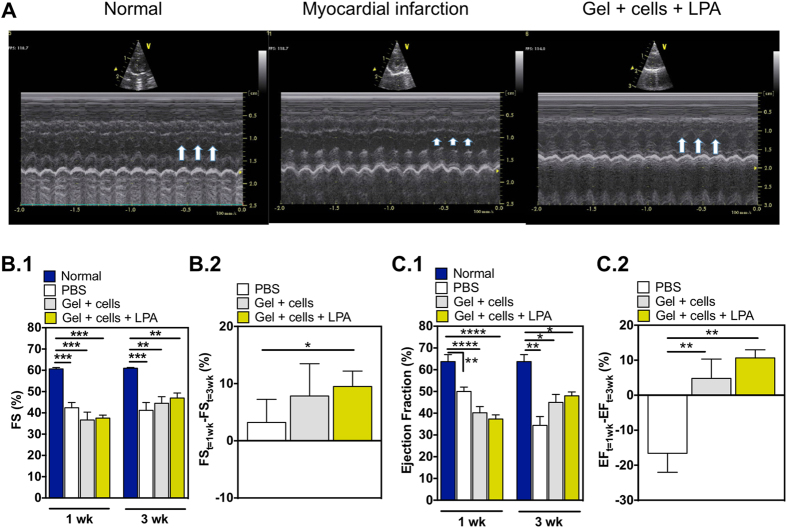
Cardiac performance after transplantation of LPA-treated CD34^+^ cells. Myocardial infarctions were induced by permanent ligation of the left anterior descending coronary artery (LAD). (**A**) Representative echocardiographic images of left ventricles before (Normal), after myocardial infarction without and with Gel + cells + LPA treatment. (**B.1**,**C.1**) Cardiac fractional shortening (**B.1**) or ejection fraction (**C.1**) in animals with (PBS, Gel + cells, Gel + cells + LPA) or without ligation of left anterior descending coronary artery (Normal). In LPA-treated CD34^+^ cells, the cells were treated with 100 μM of LPA, 1 h before transplantation. Cells (1 × 10^6^ cells) were delivered in a fibrin gel precursor solution (200 μL) into the infarcted heart of nude rats. (**B.2**,**C.2**) Cardiac fractional shortening (**B.2**) and ejection fraction (**C.2**) at week 3 was normalized by week 1. In graphs (**B**,**C**) results are presented as mean ± SEM (n = 6–10). *Denotes statistical significance: *P < 0.05, **P < 0.01, ***P < 0.001, ****P < 0.0001.

To evaluate the survival and therapeutic potential of LPA-treated CD34^+^ cells (1 × 10^6^ cells), cells were suspended in a fibrin gel precursor solution (200 μL) and injected in the infarcted heart of nude rats. Myocardial infarctions were induced by permanent ligation of the left anterior descending coronary artery (LAD). Infarcted hearts without any treatment (PBS) or treated with CD34^+^ cells suspended in a fibrin gel precursor solution were used as controls. After 2 weeks post-implantation, the functional properties of the heart were evaluated by echocardiography (Fig. 5 and Supplementary Table 3). Left ventricles of control animals had a mean fractional shortening of 42.7 ± 1.6 and 41.6 ± 3.8 at the first (week 1) and the second echocardiography evaluations (week 3 after MI procedure), and an ejection fraction of 50.0 ± 5.0 and 34.4 ± 9.1, respectively (Fig. 5). Animals treated with CD34^+^ cells encapsulated in a fibrin gel precursor solution had a mean fractional shortening of 36.7 ± 3.4 and 44.5 ± 3.0 at first and second echocardiography evaluation, and an ejection fraction of 41.5 ± 6.6 and 42.7 ± 10.0, respectively (Fig. 5). Finally, animals treated with LPA-treated CD34^+^ cells and encapsulated in a fibrin gel precursor solution had a mean fraction shortening of 37.5 ± 1.2 and 47.0 ± 2.0 at first and second echocardiography evaluation, and an ejection fraction of 37.3 ± 4.6 and 48.0 ± 4.3, respectively (Fig. 5). Because the initial fractional shortenings and ejection fractions are different between experimental groups, we used the individually normalized fractional shortenings and ejection fractions between the two measurement echocardiography events to compare the therapeutic effectiveness of the treatments. The differences between the means in the fractional shortenings were +9.5 and −1.1 in LPA-treated cells in gels and control (PBS) groups, respectively, being the differences statistically significant (*P* < 0.05). No statistical differences were observed between hearts treated with CD34^+^ cells and control (*P* > 0.05). Similar trend between groups was observed for ejection fraction. This parameter is in fact a more reliable parameter to evaluate left ventricle properties after myocardial infarction and after treatment since it evaluates the overall ventricle function rather than a section of the ventricle as in the fractional shortening. Taken together, the delivery of LPA-treated CD34^+^ cells into the infarcted heart improved cardiac fractional shortening and ejection fraction as compared to control (PBS) experimental group.

**Figure 6:**
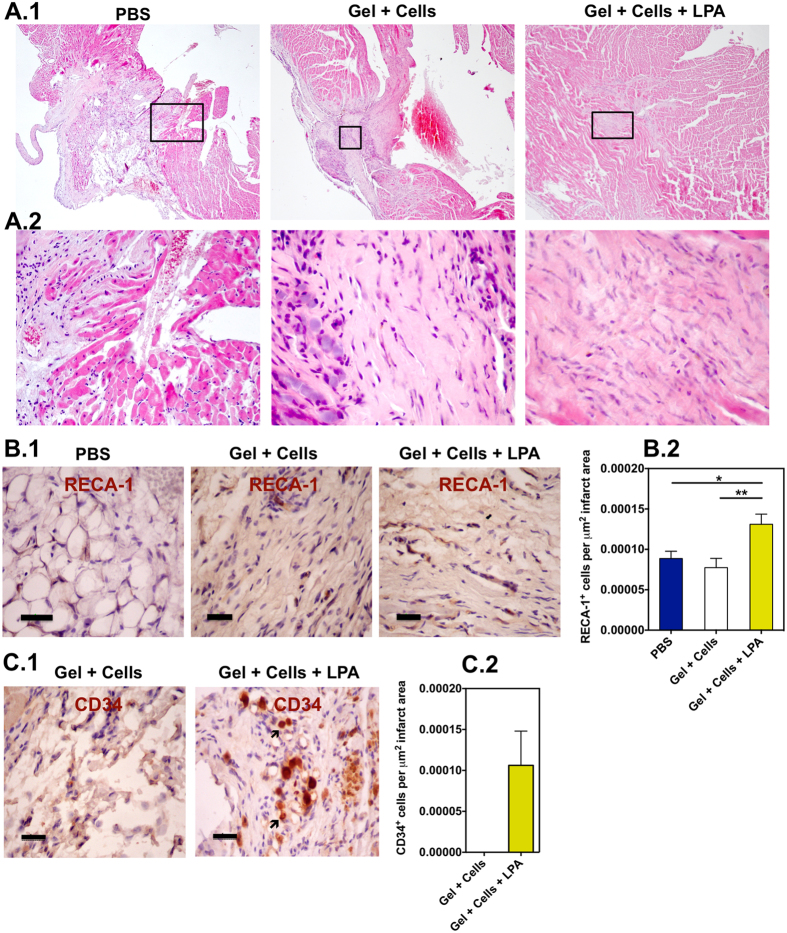
*In vivo* survival and therapeutic mechanism of LPA-treated CD34^+^ cells. (**A**) Representative transverse heart sections stained with Hematoxylin and Eosin in animals treated with PBS, fibrin gel containing CD34^+^ cells or LPA-treated CD34^+^ cells. Samples were collected at week 3 after myocardial infarction. Magnification 40× (**A.1**) and 400× (**A.2**). Images in (**A.2**) are magnifications of regions defined in A.1. (**B**) Localization (**B.1**) and number (**B.2**) of neovessels staining for rat endothelial marker RECA-1 in the infarcted area (*n* = 3–5). (**C**) Localization (**C.1**) and number (**C.2**) of human CD34^+^ cells in the heart. In (**B**,**C**) scale bar corresponds to 50 μm. *Denotes statistical significance: *P < 0.05, **P < 0.01, ***P < 0.001.

**Figure 7:**
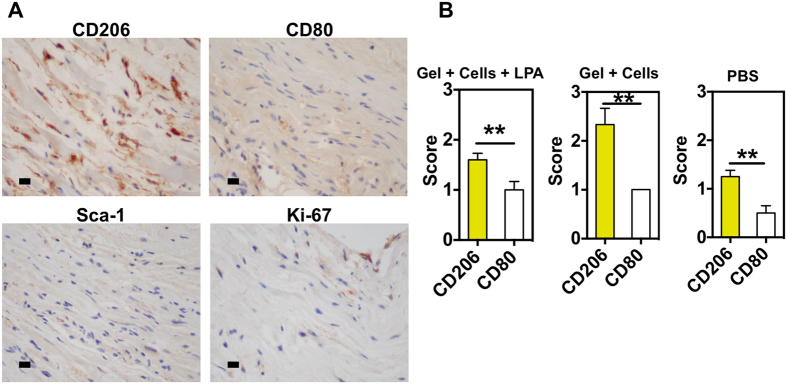
*In vivo* therapeutic mechanism of LPA-treated CD34^+^ cells. After myocardial infarction, left ventricles were treated with PBS, fibrin gel containing CD34^+^ cells or LPA-treated CD34^+^ cells. (**A**) Representative images of heart rats treated with fibrin gel containing LPA-treated CD34^+^ cells and immunostained for CD206, CD80, Ki-67 and Sca-1. (**B**) Quantification of staining for CD206 and CD80 in hearts from 3–5 different animals and more than 3 sections for each animal. Score 0, 1, 2 and 3 means that 0%, below 10%, between 10 and 50% and above 50% of the cells stained for the corresponding marker. The quantification was performed by a pathologist. *Denotes statistical significance: *P < 0.05, **P < 0.01.

To better understand the functional improvement as assessed by echocardiography, we analyzed the rat hearts at 3 weeks to evaluate collagen deposition, neovascularization, CD34^+^ cell survival, macrophage polarization (M2/M1), recruitment of primitive cardiac cells (Sca-1^+^ cells) and cell proliferation (Ki-67^+^ cells). Hematoxylin and eosin staining shows deposition of collagen in the infarct area for hearts treated with LPA-treated CD34^+^ cells or CD34^+^ cells (Fig. 6A.1). In all experimental groups, it was observed a chronic inflammation characterized by the presence of plasmocytes-plasma cells, fibroblasts as well as giant cells. Regarding neovascularization, hearts treated with gel encapsulating LPA-treated cells showed higher density of rat neovessels (RECA-1^+^ vessels) than the remaining experimental conditions (Fig. 6B.1). De novo capillary-like structures were found, mainly at the borders of the infarct but also to a lesser extent within the infarcted area. The presence of human CD34^+^ cells in the infarcted area was also assessed by immunohistochemistry. Human cells were only observed in hearts injected with LPA-treated CD34^+^ cells but not in hearts injected with untreated CD34^+^ cells (Fig. 6C.1). Hearts treated with gel encapsulating LPA-treated cells showed very low number of primitive cardiac cells (Sca-1^+^ cells) as well as negligible cell proliferation (Ki-67^+^ cells) (Fig. 7A). However, animals treated with fibrin gel containing LPA-treated CD34^+^ cells showed higher number of inflammatory cells than non-treated animals and lower number than animals treated with fibrin gel containing CD34^+^ cells (Fig. 7A,B). Although in all cases CD206^+^ macrophage cells were in higher number than CD80^+^ macrophage cells, the number of CD206^+^ cells in animals with fibrin gel containing LPA-treated CD34^+^ cells was lower than the one obtained for animals treated with fibrin gel containing CD34^+^ cells.

## Discussion

Studies have shown that CD34^+^ cells isolated from human cord blood augment the neovascularization and myocardial function in animal models of myocardial infarction^6^,^10^,^11^. These cells may be a suitable source of cell therapy for patients that underwent a myocardial infarction and had low number of CD34^+^ cells (such as aged patients^46^) or impaired CD34^+^ function (such as patients with diabetes)^47^. In this study, we show that LPA-treated CD34^+^ cells have improved survival in ischemia conditions both *in vitro* and *in vivo*. We elucidate the mechanism underlying the survival process. Finally, we show that LPA-treated CD34^+^ cells delivered in fibrin gels, improve cardiac fractional shortening and ejection fraction compared to non-treated hearts (treated with PBS).

Several studies indicate that LPA pro-survival effect is mediated by G-protein-coupled receptors (GPCRs; i.e., LPA1, LPA2, LPA3, LPA4 and LPA5)^14^,^15^,^16^. These LPA receptors are coupled to heterotrimeric G-proteins such as G_αi_, G_αq_ and G_α12/13_. In case of mesenchymal stem cells^14^ and Schwann cells^16^, LPA1 mediates the pro-survival effect. The LPA pro-survival effect is mediated by the activation of ERK pathway in fibroblasts^15^ and both Akt and ERK pathways in intestinal epithelial cells^41^, mesenchymal stem cells^14^,^43^, Schawnn cells^48^ and hepatocytes^49^. Our results show that the pro-survival effect of LPA in CD34^+^ cells is mainly mediated by PPAR-γ. Previous studies^50^, as well as the results obtained in the current work, show that CD34^+^ cells express PPAR-γ. PPAR-γ is a nuclear receptor that binds to specific DNA regulatory elements forming heterodimers with the retinoid X receptor^50^. Since LPA is a phospholipid, it can cross the cell membrane and activates intracellular receptors^29^,^30^. LPA-induced PPAR-γ activation has been reported before to mediate the effect of LPA in cell migration^36^ but, to the best of our knowledge, was never evaluated in the context of cell survival. Mesenchymal stem cells do not express PPAR-γ^50^, which might explain their insensitivity to this signaling pathway.

The activation of PPAR-γ in CD34^+^ cells activates both pro-survival pathways ERK and Akt and inhibits cell apoptosis decreasing Bax and caspase 9 expression. These results are in line with the activation of both pro-survival pathways in cardiomyocytes after the activation of PPAR-γ signaling^51^ and the anti-apoptotic role of LPA on intestinal epithelial cells and T cells by preventing translocation of the apoptosis regulator Bax from cytosol to mitochondria^41^.

The mechanisms underlying the proliferative effect by LPA are diverse and depend on cell type and culture conditions. Activation of GPCR-dependent (LPA2 and LPA3 receptors^52^ and downstream G_α12_^53^) or GPCR-independent^54^ signaling pathways have been documented to mediate the proliferation of LPA-treated cells. Downstream signaling pathways include the activation of ERK, protein kinase C, Akt, β-catenin, Ca^2+^ mobilization and Rho A pathways^52^,^53^,^55^. In the present work, LPA induces CD34^+^ cell proliferation under hypoxia and serum-deprived conditions. Cell cycle analyses after 24 h of hypoxia show that LPA-treated cells enter S phase and consequently G2M phase faster than non-treated cells. Further experiments are needed to evaluate whether cell proliferation is mediated by PPAR-γ signaling. It is important to note that PPAR-γ agonists such as adiponectin and thiazolidinediones have been described to increase the number of CD34^+^ cells^56^.

LPA is unable to maintain the undifferentiated state of CD34^+^ cells. Our results show that with or without LPA treatment, CD34^+^ cells spontaneously differentiate into mast cells and cells of myeloid lineage (essentially neutrophils and monocytes). Previously, it was shown that LPA (5 μM) increased the differentiation of human mononuclear cells into mast cells when the cells were cultured in the presence of stem cell factor (SCF), IL-6 and IL-10 for 2 weeks^57^. The capacity of LPA to enhance erythropoiesis in cord-blood-derived human hematopoietic stem cells has also been demonstrated^21^. This effect was mediated by the activation of LPA3 receptor. The differentiation protocol consisted in 3 steps, and involved different culture media supplemented with SCF, erythropoietin, IL-3, vascular endothelial growth factor and insulin-like growth factor II. Finally, during the preparation of this work, it was shown that LPA (1 μM) enhanced myeloid but not lymphoid differentiation of CD34^+^ cells^20^. These studies have been carried out in normoxia for 4 weeks, involved the use of feeder layers (OP9 stromal cell line) and culture media supplemented with growth factor combinations permissive for both myeloid and lymphoid differentiation (thrombopoietin, Flt3 ligand and IL-7). In the present work, we have performed our studies in hypoxia (initial 24 h) followed or not by 5 days of culture in normoxia, the cells were cultured in the absence of feeder layers, and culture media (X-Vivo medium) without any growth factor/cytokine supplements. Therefore, the differences observed between the results in our work and the previous ones are due to differences in cell culture conditions. It should be noted that our goal was to evaluate CD34^+^-lineage commitment after culture in hypoxia and serum-deprived conditions and not the effect of LPA in CD34^+^ differentiation protocols having different differentiating agents.

LPA modulates differently cytokine release in CD34^+^ cells and CD34^+^ cells encapsulated in fibrin gels. It is recognized by several studies that the regenerative effect of transplanted CD34^+^ cells in myocardium after infarction is mediated by its paracrine effect^4^,^58^,^59^. Recruitment of monocytes/macrophages was significantly enhanced in human CD34^+^-transplanted murine hearts after infarction and this effect seems to be mediated by human cytokines MCP-1 and IL-8^45^. In addition, blocking vascular endothelial growth factor secreted by human CD34^+^ cells abolished the functional (ejection fraction) improvement exerted by the stem cells in the murine infarcted heart^59^. Exosomes secreted by CD34^+^ cells may also represent a significant component of the paracrine effect of stem cells and mediate angiogenesis processes^58^. Our results indicate that LPA-treated CD34^+^ cells secreted higher levels of IL-6, IL-8 and G-CSF during the first 12 h than non-treated cells. However, at time 24 h, LPA-treated CD34^+^ cells secreted lower levels of all the measurable cytokines such as G-CSF, IFN-γ, IL-6, IL-8, IL-17, MCP-1, MIP-1β, and TNF-α than non-treated cells. Importantly, we demonstrated that the secretion profile of LPA-treated CD34^+^ cells was mediated by PPAR-γ signaling pathway. Previous studies have shown that LPA induced the expression of MCP-1 and IL-8 in human umbilical vein endothelial cells (HUVECs) through a G_i,o_-, Rho- and nuclear factor (NF)κB-dependent mechanisms^60^. In a separate study, LPA (from 1 to 25 μM) enhanced IL-8 and MCP-1 expression in both EAhy926 (endothelial cell line) and HUVECs through G_i,o_-, LPA1-, LPA3- and PPAR-γ-dependent pathways^36^. In both studies, the secretion of MCP-1 and IL-8 peaked before 12 h of cell treatment with LPA. Further, LPA induced the secretion of IL-6 and IL-8 by mature dendritic cells^61^. In the current work, the demonstration that LPA-treated CD34^+^ cells secrete higher levels of IL-6 and IL-8 during the first 12 h might be beneficial for the recruitment of monocytes/macrophages, key cellular players in the regeneration process^45^.

Because LPA-treated CD34^+^ cells were injected in a myocardial infarction animal model in a fibrin gel, we evaluated their secretome. Our results highlight significant differences in the secretome of LPA-treated CD34^+^ cells in suspension or encapsulated in fibrin gels. First, encapsulation of the cells in fibrin gels changed the magnitude and profile of cytokines secreted by non-treated and LPA-treated CD34^+^ cells. It is unclear the impact of these changes in the regenerative potential of CD34^+^ cells in the heart. It is likely that the differences in the secretome of CD34^+^ cells in suspension and encapsulated in fibrin gels are due to cell-matrix interactions, but further studies should be done in the near future to clarify this issue. Second, IL-13 was only observed in cells (either treated or not with LPA) encapsulated in fibrin gels. IL-13 is a protector of vascular injury having a regulatory role in the biosynthesis of fibrinogen^62^. IL-13 also stimulates the migratory properties of hematopoietic progenitor cells^63^. It is interesting to note that a very recent study has shown that IL-13 is important for cardiac regeneration^64^. Therefore, we speculate that the secretion of IL-13 induced in CD34^+^ cells encapsulated in fibrin gel might be important for the regeneration of the heart. Third, although the secretory ability of the encapsulated cells is reduced *in vitro*, in conditions that there is no degradation of the extracellular matrix, it is expected that *in vivo* conditions, in the context of a rich metalloprotease environment, the secretory ability will be different. Further *in vivo* testing needs to be performed to clarify this issue.

LPA-treated CD34^+^ cells entrapped in fibrin gels survived for 3 weeks. Although some studies showed that CD34^+^ cells can persist in the heart for up to 12 months following injection into the peri-infarct zone of several combined immunodeficient mice^59^, other studies have shown that most of CD34^+^ cells die after few days of transplantation in the infarcted heart due to ischemia^6^. Because paracrine effects mediate the regenerative properties of CD34^+^ cells, it is important to maintain their survival in the heart. Here, we show that LPA-treated CD34^+^ cells have enhanced survival in the infarcted heart relatively to non-treated CD34^+^ cells. Previous studies have used several strategies to increase cell survival such as cell conditioning (ex: heat shock, hypoxic pre-conditioning, hypoxia inducible factor-1, erythropoietin), anti-apoptotic pathways (Rho-associated kinase inhibition, TGF-β_2_ treatment, p38 MAPK inhibition), pro-survival cocktails (Akt and Bcl overexpression, cyclosporine, ZVAD-fmk, insulin-like growth factor-1), co-transplant of cells (mesenchymal cells, fibroblasts, endothelial progenitor cells), improving cell retention (ex: injectable hydrogels), among others^65^. Unfortunately, very few studies have used pro-survival strategies for CD34^+^ cells. These included the use of hydrogels to protect CD34^+^ cells from anoxia-induced apoptosis^66^, co-culture with endothelial cells^9^, treatment with prostaglandin E_2_ 67, and activation of NF-κB/Rel transcription factors by amifostine^68^. In the current work, we have used a cost effective biomolecule present in the human body that shows higher pro-survival activity than known CD34^+^ cell pro-survival molecules including prostaglandin E_2_ 67. The pro-survival activity of LPA has been recently demonstrated in mesenchymal stem cells transplanted in myocardium after infarct^40^.

Hearts treated with fibrin gel containing LPA-treated CD34^+^ cells as well as in the other experimental conditions (non-treated hearts or hearts treated with CD34^+^ cells) showed low levels of cardiac primitive cells (Sca-1^+^ cells) as well as cell proliferation at week 3. However, hearts treated with fibrin gel containing LPA-treated CD34^+^ cells or non-treated CD34^+^ cells show higher number of macrophage cells, and in particular M2 macrophages (CD206^+^ cells), than non-treated hearts. Previous studies have shown that optimum outcome after myocardial infarction depends on a coordinated healing response that balances debris removal by macrophages. M1 macrophages promote inflammation and ECM destruction while M2 macrophages facilitate ECM reconstruction, cell proliferation, and angiogenesis^69^. Therefore, our results indicate that the healing response is superior in hearts treated with CD34^+^ cells than non-treated hearts.

## Conclusions

In this work, we show that LPA enhances the survival and modulates the secretion of pro-inflammatory cytokines in CD34^+^ cells cultured in ischemic conditions, i.e., in hypoxia and serum-deprivation cultured conditions. In contrast to other cell types, including mesenchymal stem cells, CD34^+^ cell survival and cytokine secretion are mainly mediated by the activation of PPAR-γ and not GPCR signaling pathways. The activation of PPAR-γ seems to induce both the activation of Akt and ERK signaling pathways and the inhibition of mitochondrial apoptotic pathway (i.e. decreasing the expression of *BAX* and *CASP9*). Under hypoxia and serum-deprivation culture conditions, LPA induces CD34^+^ cell proliferation, but is unable to maintain their undifferentiated state. In addition, during the first 12 h of LPA treatment, CD34^+^ cells secrete higher levels of IL-6, IL-8 and G-CSF pro-inflammatory cytokines than non-treated cells; however, at time 24 h, LPA-treated CD34^+^ cells secrete lower levels of all the measurable cytokines such as G-CSF, IFN-γ, IL-6, IL-8, IL-17, MCP-1, MIP-1β and TNF-α than non-treated cells. Our *in vivo* results show that LPA-treated CD34^+^ cells have superior survival than non-treated cells, hence being able to contribute to the preservation of cardiac function after infarction.

## Materials and Methods

### Isolation of CD34^+^ cells from UCB

All human umbilical cord blood samples were collected from donors, who signed an informed consent form, in compliance with Portuguese legislation. The collection methods were authorized and carried out in accordance with the approved guidelines and regulations by the ethical committee of Maternity Daniel de Matos (Coimbra) and Hospital Infante D. Pedro (Aveiro). The samples were stored in sterile bags containing 35 mL of citrate-phosphate-dextrose anticoagulant solution. CD34^+^ cells were isolated from mononuclear cells, obtained from UCB samples after Ficoll/Histopaque-1077 Hybri Max (Sigma-Aldrich, Missouri, USA) density gradient separation. CD34^+^ cells were positively selected (2 times) using the mini-MACS immunomagnetic separation system (Miltenyi Biotec, Bergisch Gladbach, Germany), according to the manufacturer’s recommendations. CD34^+^ cells were immediately used for cell encapsulation studies or *in vivo* experiments without further treatment. The cells isolated were above 95% of purity for CD34 antigen as confirmed by FACS.

### Cell survival analyses with different drugs

UCB CD34^+^ cells were suspended in X-Vivo medium (Lonza, Basel, Switzerland; 1 × 10^6^ cells/mL, i.e., 2 × 10^5^ cells per well of a 96-well plate containing 200 μL of X-Vivo medium) supplemented or not with one of the following drugs: Navitoclax (ABT-263), 10 μM in 0.01% DMSO X-Vivo, Selleckchem, Germany; Nebivolol, 10 μM in 0.01% DMSO X-Vivo, Selleckchem; INO1001, 10 μM in 0.01% DMSO X-Vivo, Selleckchem; Irbesartan, 10 μM in 0.01% DMSO X-Vivo, Selleckchem; VX-702, 10 μM in 0.01% DMSO X-Vivo, Selleckchem; recombinant human erythropoietin, 0.25 UI/mL in X-vivo, Sigma-Aldrich, Missouri, USA; 16,16-Dimethyl Prostaglandin E2, 10 μM in 0.01% DMSO X-Vivo, Calbiochem, California, USA and LPA, 10 μM in X-vivo, Sigma-Aldrich. The cells were incubated for 1 h in normoxia conditions (21% O_2_, 5% CO_2_, 37 °C) followed by their incubation in a hypoxia chamber for 24 h (0.5% of O_2_ and 5% of CO_2_), in the presence or absence of pharmacological drugs. Cell survival was determined by annexin V/propidium iodide (PI) (Invitrogen, California, USA) staining, using the FACS Calibur cytometer and CellQuest software (BD Biosciences, New Jersey, USA). Annexin V is a phospholipid-binding protein with specificity for phospatidyl serine, one of the earliest makers of cellular transition to an apoptotic state. This phospholipid is translocated from the inner to the outer leaflet of the plasma membrane^70^. PI passes through the membranes of necrotic cells and intercalates into nucleic acids due to decreased integrity of the plasma and nuclear membranes^71^. CD34^+^ cells (200.000 cells) were suspended in annexin binding buffer (200 μL) supplemented with Annexin V (3.5 μL). The tubes were incubated for 10 min in the dark and then 2 μL of a propidium iodide stock solution (1 mg/mL) was added before the samples were analyzed by flow cytometry. Approximately 20.000 events were collected in each analysis.

### Cell survival analyses in hypoxia and normoxia conditions

UCB CD34^+^ cells were suspended in X-Vivo medium, i.e., 2 × 10^5^ cells per well of a 96-well plate containing 200 μL of X-Vivo medium with or without LPA 100 μM (in 0.01% DMSO X-Vivo). The cells were incubated in normoxia (21% O_2_, 5% CO_2_, 37 °C) conditions for 1 h. Then, the plates were incubated in hypoxic (0.5% O_2_, 5% CO_2_, 37 °C) or normoxic (21% O_2_, 5% CO_2_, 37 °C) conditions for 24 h. LPA was maintained in the culture medium during the 24 h period. Cell survival was determined by annexin V/propidium iodide (PI) as described before.

### Cell survival analyses: effect of hypoxia/normoxia time

UCB CD34^+^ cells were suspended in X-Vivo medium, i.e., 2 × 10^5^ cells per well of a 96-well plate containing 200 μL of X-Vivo medium with or without LPA 100 μM (in 0.01% DMSO X-Vivo). The cells were incubated in normoxia (21% O_2_, 5% CO_2_, 37 °C) conditions for 1 h. Then, the plates were incubated in hypoxia (0.5% O_2_, 5% CO_2_, 37 °C) for 1 or 3 days, or in hypoxia for 1 day followed by 3 days in normoxia. LPA was maintained in the culture medium during the 24 h period. Cell survival was determined by annexin V/propidium iodide (PI) as described before.

### Cell survival analyses: effect of LPA concentration

UCB CD34^+^ cells were suspended in X-Vivo medium, i.e., 2 × 10^5^ cells per well of a 96-well plate containing 200 μL of X-Vivo medium supplemented with LPA 1, 5, 10 and 100 μM (in 0.01% DMSO X-Vivo). The cells were incubated in normoxia (21% O_2_, 5% CO_2_, 37 °C) conditions for 1 h. Then, the plates were incubated in hypoxic (0.5% O_2_, 5% CO_2_, 37 °C) conditions for 24 h. LPA was maintained in the culture medium during the 24 h period. Cell survival was determined by annexin V/propidium iodide (PI) as described before.

### Cell survival: mechanism

UCB CD34^+^ cells were suspended in X-Vivo medium, i.e., 2 × 10^5^ cells per well of a 96-well plate containing 200 μL of X-Vivo medium supplemented with one of the following antagonists: MK2206 (3 μM, Selleckchem), PD98059 (60 μM, CaymanChem, Michigan, USA), GW9662 (50 μM, Calbiochem), Y-2762/ROCK (50 μM, Calbiochem), Ki16425 (10 μM, Selleckchem) or 1-Bromo-3(S)-hydroxy-4-(palmitoyloxy)butyl]phosphonate (BrP-LPA) (1.5 μM, Echelon Biosciences Inc.). After 10 min of incubation, LPA 100 μM (in 0.01% DMSO X-Vivo) was added and the cells incubated in normoxia (21% O_2_, 5% CO_2_, 37 °C) conditions for 1 h. Then, the plates were incubated in hypoxic (0.5% O_2_, 5% CO_2_, 37 °C) conditions for 24 h. Antagonists and LPA were maintained in the culture medium during the 24 h period. Cell survival was determined by annexin V/propidium iodide (PI) as described before.

### Cell survival: gene analyses (*VEGFA*, *LPARs*, *BAX*, *CASP9, CCNG2*)

UCB CD34^+^ cells were suspended in X-Vivo medium, i.e., 2 × 10^5^ cells per well of a 96-well plate containing 200 μL of X-Vivo medium with or without LPA 100 μM (in 0.01% DMSO X-Vivo). The cells were incubated in normoxia (21% O_2_, 5% CO_2_, 37 °C) conditions for 1 h. Then, the plates were incubated in hypoxia (0.5% O_2_, 5% CO_2_, 37 °C) for 4, 8, 12 or 24 h. LPA was maintained in the culture medium during the 24 h period. Cells were collected and RNA extracted according to the protocol below. In case of cells encapsulated in fibrin gels, the constructs were centrifuged and incubated with lysis buffer and glass beads (Advanced Tecnologies) until the whole gel was depolymerized. RNA was then extracted according to the protocol below.

### CD34^+^ cell proliferation analyses

UCB CD34^+^ cells were suspended in X-Vivo medium, i.e., 2 × 10^5^ cells per well of a 96-well plate containing 200 μL of X-Vivo medium with or without LPA 100 μM (in 0.01% DMSO X-Vivo). The cells were incubated in normoxia (21% O_2_, 5% CO_2_, 37 °C) conditions for 1 h. Then, the plates were incubated in hypoxia (0.5% O_2_, 5% CO_2_, 37 °C) for 1 day followed by 6 days in normoxia. LPA was maintained in the culture medium during the assay. Cell count was performed with a hemocytometer.

### Cell cycle analyses by flow cytometry

UCB CD34^+^ cells were suspended in X-Vivo medium, i.e., 2 × 10^5^ cells per well of a 96-well plate containing 200 μL of X-Vivo medium with or without LPA 100 μM (in 0.01% DMSO X-Vivo). The cells were incubated in normoxia (21% O_2_, 5% CO_2_, 37 °C) conditions for 1 h and then in hypoxia (0.5% O_2_, 5% CO_2_, 37 °C) for 1 day. Cells were fixed by adding cold 70% (w/v) ethanol, drop by drop to the tube wall while vortexing to avoid the formation of cell aggregates. Cells were kept at −20 °C until they were further processed for analysis. For staining, cells were washed once with PBS, incubated for 30 min at room temperature with a solution containing propidium iodide (PI, 20 μg/mL, Sigma), 200 μg/mL DNAse-free RNAse and 0.1% (v/v) Triton in PBS and analyzed with a FACS Calibur (BD Biosciences). CellQuest software (BD Biosciences) was used to quantify the percentage of the different cell cycle phases.

### Cell differentiation analyses

UCB CD34^+^ cells were suspended in X-Vivo medium, i.e., 2 × 10^5^ cells per well of a 96-well plate containing 200 μL of X-Vivo medium with or without LPA 100 μM (in 0.01% DMSO X-Vivo). The cells were incubated in normoxia (21% O_2_, 5% CO_2_, 37 °C) conditions for 1 h. Then, the plates were incubated in hypoxia (0.5% O_2_, 5% CO_2_, 37 °C) for 1 day or incubated in hypoxia for 1 day followed by 6 days in normoxia. In both cases, cells were characterized by FACS using a 4-tube characterization protocol. The following CD34^+^ sub-populations were identified by a FACS Canto II flow cytometer (BD Biosciences): uncommitted (more immature) precursors, neutrophil precursors, B cell precursors, monocytic precursors, plasmacytoid dendritic cells precursors, erythroid precursors, basophil precursors and mast cell precursors. The markers used are listed in Supplementary Tables 1 and 2. FACSDiva (BD Biosciences) and Infinicyt™, v.1.5 (Cytognos SL, Salamanca, Spain) software’s were used for acquisition and analysis, respectively.

### Preparation of fibrin gels

Fibrin gels were formed by crosslinking of fibrinogen (Sigma-Aldrich) in the presence of thrombin (Sigma-Aldrich), as reported before by us^9^. Briefly, the fibrinogen solution was prepared by dissolving human fibrinogen in Tris-buffered saline (TBS) (Sigma-Aldrich), pH 7.4 (20 mg/mL), and then sterilized by filtering through 0.22 μm syringe filter (Acrodisc, Washington, USA). Fresh thrombin solutions were prepared by dissolving human thrombin in TBS at pH 7.4 at a concentration of 50 U/mL. Fibrin gels (200 μL for *in vivo* and 50 μL for *in vitro*) were prepared by mixing three different components: fibrinogen (10 mg/mL), CaCl_2_ (Merck, New Jersey, USA) (2.5 mM) and thrombin (2 U/mL). This solution was allowed to polymerize at 37 °C and 95–98% relative humidity.

### Cell survival: CD34^+^ cells encapsulated in fibrin gel

UCB CD34^+^ cells encapsulated in a fibrin gel (i.e. 2 × 10^5^ cells per 50 μL of gel) were suspended in X-Vivo medium (200 μL) with or without LPA 100 μM (in 0.01% DMSO X-Vivo). The cells were incubated in normoxia (21% O_2_, 5% CO_2_, 37 °C) conditions for 1 h and then in hypoxia (0.5% O_2_, 5% CO_2_, 37 °C) for 1 day. At the end, the survival of the cells was evaluated by fluorescence microscopy using AnnexinV/PI staining.

### Cytokine secretion analyses

Cell culture supernatants were evaluated for the presence and concentrations of cytokines using a Bio-Plex Pro Human Cytokine 17-Plex Panel Assay (Bio-Rad, California, USA), according to manufacturer’s instructions, in a Bio-Plex 200 System (Bio-Rad). The human group I 17-plex panel consisted of the following analytes: interleukin-1β (IL-1β), IL-2, IL-4, IL-5, IL-6, IL-7, IL-8; IL-10, IL-12(p70), IL-13, IL-17, granulocyte colony-stimulating factor (G-CSF), granulocyte/macrophage colony-stimulating factor (GM-CSF), interferon-γ (IFN-γ), monocyte chemotactic protein (monocyte chemotactic activating factor [MCP-1 (MCAF)], macrophage inflammatory protein-β (MIP-1β) and tumor necrosis factor-α (TNF-α). Cell culture supernatants were collected, centrifuged to remove precipitates and frozen at −80 °C. A standard range of 0.2 to 3.200 pg/mL was used. Samples and controls were run in triplicates, standards and blanks in duplicates.

### Caspase activity

Cells in suspension and cells encapsulated in fibrin gels were prepared as before. After 24 h in hypoxia, the amount of apoptotic cells was determined using Caspase-Glo^®^ 9 assay (Promega, WI, USA) according to manufacturer’s instructions. The assay depends on the generation of a “glow-type” luminescent signal produced by the luciferase reaction between luminogenic caspase-9 substrate and caspase-9 enzyme present in the cells. Suspended cells and cells encapsulated in fibrin gels were cultured as described in previous sections for 24 h. Equal volumes of Caspase-Glo^®^ reagent and cell culture medium were mixed in each well and incubated at room temperature for 2 min on an orbital shaker to induce cell lysis. Then, the cells were transferred into opaque-walled multiwell plates and luminescence measurements were done using a LUMIstar Luminometer (BMG LABTECH, Germany). The medium without cells was used as a blank control.

### Quantitative reverse transcription-polymerase chain reaction (qRT-PCR) analysis

Total RNA in cells was extracted by using the RNeasy Mini Kit (Qiagen, Hilden, Germany), according to manufacturer’s instructions. In all cases, cDNA was prepared from 1 μg total RNA using Taqman Reverse transcription reagents (Applied Biosystems, California, USA). Quantitative PCR (qPCR) was performed using Power SYBR Green PCR Master Mix (Applied Biosystems, California, USA) and the detection was carried out in a 7500 Fast Real-Time PCR System (Applied Biosystems). Quantification of target genes was performed relatively to the reference human GAPDH gene: 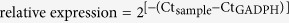. The mean minimal cycle threshold values (Ct) were calculated from four independent reactions. Primer sequences are published as supporting information (Supplementary Table 4).

### Myocardial infarction animal model

Experiments were performed on 20 adult male Rowett nude rats (Crl:NIH-*Foxn1*^*rnu*^) (Charles River Laboratories International) weighing between 180 and 250 gr. In accordance to the Directive 2010/63/EU rats were acclimatized for two weeks before animal procedures, housed in groups of 2 in individual ventilated cages (IVC) type III rat cages (Tecniplast SRL, Italy) on enriched corn cob bedding with 65 air changes per hour, ambient temperature between 21–22 °C and a relative humidity of 40–60% and an artificial light dark cycle of 12/12 hours. The animals had free access to water and standard diet *ad libitum*. Methods involved in this project were authorized and carried out in accordance with the approved guidelines and regulations by the national animal welfare body from Direção Geral de Alimentação e Veterinária (DGAV). Surgery instruments were sterilized by autoclave. Rowett nude rats (Crl:NIH-*Foxn1*^*rnu*^) were anaesthetized with ketamine (75 mg/kg, IP) and dexmedetomidine (0.375 mg/kg, IP) and placed in supine position for tracheal intubation. A 16-Gauge intravenous catheter with a 70 mm long wire stylet with a 30 mm hub was used as an endothreacheal tube. The catheter hub was connected to the respirator and rats were mechanical ventilated. Anaesthesia was afterwards maintained by 1–2% isoflurane in balanced oxygen. The abdomen and anterior chest were scrubbed with betadine and wiped with 70% alcohol (with several cycles of betadine scrub followed by alcohol rinse and application of betadine solution). The heart was approached either by a transverse abdominal incision (diaphragmatic incision) with the animal’s back gently extended over a soft towel or laterally via the intercostal space 4–5. A small diaphragmatic incision was made to create a pericardial window. A 5 mm incision was made in the pericardium with an 11–0 scalpel. Myocardial infarction was induced by permanent ligation of the left anterior descending coronary artery with a 6–0 Proline suture, 2–3 mm below the origin of the artery. Pallor and regional wall motion abnormality of the left ventricle confirmed occlusion. The pericardium was left open or removed to avoid tamponade. The pleural space was evacuated with an 18-gauge sterile needle and 3-ml syringe following closure of the pleural cavity. Abdominal wall and subcutaneous tissue were closed with Vicryl 4–0 followed by a subcuticular closure with Vicryl 4–0. The animal was extubated and then allowed to recover. Each animal was maintained during surgical procedures and recovery under warming pads until awake and able to ambulate. Two or three days after recovery from this procedure, animals underwent echocardiographic evaluation under ketamine/midazolam anesthesia. Echocardiographic evaluation was performed by using a Vivid i ultrasound equipment and a 10S probe (GE healthcare). Animals meeting the echocardiographic inclusion criterion (fractional shortening below 50%) were stratified into one of 3 groups. One week after MI procedure the rats were subjected to a second thoracotomy followed by direct injection of 200 μL of therapeutic agent using Micro Fine Plus 30-gauge needle (BD Bioscience) into the myocardium. Two weeks after implantation, the surviving rats (3 weeks after MI) were submitted to a second echocardiographic evaluation under ketamine/midazolam anesthesia and fractional shortening was accessed. At week 4 after MI procedure (3 weeks after implantation) animals were euthanized with lethal dose of anesthetics. The hearts were harvested and processed for histological analysis. One group of heart was placed Optimal Cutting Temperature (OCT) and immediately frozen and stored at −80 °C. Other group was stored in 4% paraformaldehyde (with 10% formalin) and stored at room temperature.

### Histology and immunohistochemistry analyses

The hearts were explanted from the rats and frozen at −80 °C. For further processing they were thawed in formaldehyde (BDH Prolabo, 4%) and embedded in paraffin (Merck, Histosec) which was followed by Microtome sectioning (Leica). Transverse sections of 10 μm were placed on silane-coated slides (Leica) for Immunostaining or uncoated slides for H&E and Masson’s Trichrome staining and allowed to dry overnight. After deparaffinization and rehydration, samples were stained with H&E solution (Sakura- Tissue- Tek® DRS™) or Masson´s Trichrome. Labelled Streptavidin and Biotin methodology was used for immunostainning, following sample pre-treatment for antigen unmasking. CD34^+^ antigen unmasking was achieved by slide treatment with EDTA for 20 min while RECA-1 antigen unmasking was achieved by treatment with Pronase E for 10 min at room temperature. Endogenous peroxidase activity was quenched using 15 min incubation in 3% diluted hydrogen peroxide (H_2_O_2_). For blocking non-specific binding, Ultra V Block (Ultra Vision Kit; TP-015-HL) was applied to the sections and they were incubated with primary antibodies, anti-Human CD34^+^ (Dako, 1:25, 30 min) and anti-rat RECA-1 (Abcam, 1:200, 30 min). The slides were counterstained with hematoxylin, dehydrated and mounted.

In case of CD206, Sca-1, CD80, and Ki-67 immunohistochemistry, the antigens were unmasked by enzyme treatment (10 min, Pronase E, in case of CD206), bond epitope retrieval solution 1 (pH 6, 20 min, in case of Sca-1) or bond epitope retrieval solution 2 (pH 8, 20 min, in case of CD80 or Ki-67). Endogenous peroxidase activity was quenched using 5 min incubation in 3% (v/v) diluted H_2_O_2_. Immunohistochemical staining was performed with Bond Polymer Refine Detection™ (DS9800; Leica Biosystems, Newcastle Ltd, United Kingdom) according to manufacturer’s instructions on BondMax. Sections were incubated with anti-rat primary antibodies, CD206 (Santa Cruz, 1:100, 30 min), CD80 (Santa Cruz, 1:75, 30 min), Sca-1 (Abcam, 1:300, 30 min) and Ki-67 (Santa Cruz, 1:50, 30 min). After staining, the slides were counterstained with hematoxylin (5 min), dehydrated and mounted. In parallel, known positive and negative controls were used. The intensity of the staining was graded semi-quantitatively on a four point scale: 0 − negative; 1+ < 10%, 2+ 10–50%, 3+ > 50% percentage of immunostained cells.

### Statistical analyses

Statistical analyses were performed using GraphPad PRISM software. One-way ANOVA with post Newman-Keuls testing or Dunnett multiple comparison testing was used to examine statistical differences between multiple groups. Unpaired t-test was used to examine statistical differences between two independent groups.

## Additional Information

**How to cite this article**: Kostic, I. *et al*.Lysophosphatidic acid enhances survival of human CD34^+^ cells in ischemic conditions. *Sci. Rep*.**5**, 16406; doi: 10.1038/srep16406 (2015).

## Supplementary Material

Supplementary Information
