# Supplementary material for: Lysophosphatidic acid enhances survival of human CD34+ cells in ischemic conditions
Source: Sci Rep. 2015 Nov 10;5:16406. doi: 10.1038/srep16406 (PMC4639756; doi:10.1038/srep16406)
Supplement: Supplementary Information [file srep16406-s1.doc]

**Lysophosphatidic acid enhances survival of human CD34^+^ cells in ischemic conditions**

Ivana Kostic^1,2*^, Isabel Fidalgo-Carvalho^1*^, Sezin Aday^1,2^, Helena Vazão^1,2^, Tiago Carvalheiro^3^, Mário Grãos^1^, António Duarte^5^, Carla Cardoso^6^, Lino Gonçalves^5^, Lina Carvalho^7^, Artur Paiva^3^, Lino Ferreira ^1,2,#^

^1^Biocant, Cantanhede, Portugal, ^2^CNC-Center for Neuroscience and Cell Biology, University of Coimbra, Coimbra, Portugal, ^3^Portuguese Institute for Blood and Transplantation, IP, ^5^Department of Cardiology, Coimbra University Hospital Center & Faculty of Medicine University of Coimbra, Coimbra, Portugal, ^6^Crioestaminal, Cantanhede, Portugal, ^7^Department of Anatomical Pathology, University Hospital of Coimbra, Coimbra, Portugal.

*Authors contributed equally.

**^#^Corresponding author:**

Lino Ferreira

Center for Neurosciences and Cell Biology

University of Coimbra

Largo Marques de Pombal

3004-517 Coimbra

E-mail: lino@biocant.pt

| **Fluorescence** | **FITC** | **PE** | **PerCPCy 5.5** | **PECy7** | **APC** | **APC-H7** | **PB** | **KrO** |
| --- | --- | --- | --- | --- | --- | --- | --- | --- |
| **Tube 1** | **CD71**  Clone: M-A712  BD Pharmingen | **CD64**  Clone: 22  Beckman Coulter | **CD34**  Clone:8G12  BD Biosciences | **CD117**  Clone: 104D2D1  Beckman Coulter | **CD36**  Clone: CB38  BD Pharmingen | **HLA-DR**  Clone:G46-6  BD Biosciences | **CD44**  Clone: IM7  Biolegend | **CD45**  Clone: J.33  Beckman Coulter |
| **Tube 2** | **CD35**  Clone: E11  BD Pharmingen | **CD123**  Clone: 9F5  BD Biosciences | **CD34**  Clone:8G12  BD Biosciences | **CD117**  Clone: 104D2D1  Beckman Coulter | **CD133**  Clone: 293C3  Miltenyi Biotec | **HLA-DR**  Clone:G46-6  BD Biosciences | **CD44**  Clone: IM7  Biolegend | **CD45**  Clone: J.33  Beckman Coulter |
| **Tube 3** | **CD61**  Clone: SZ21  Beckman Coulter | cy**CD203c**  Clone: 97A6  Beckman Coulter | **CD34**  Clone:8G12  BD Biosciences | **CD117**  Clone: 104D2D1  Beckman Coulter | - | **HLA-DR**  Clone:G46-6  BD Biosciences | **CD44**  Clone: IM7  Biolegend | **CD45**  Clone: J.33  Beckman Coulter |
| **Tube 4** | **CD35**  Clone: E11  BD Pharmingen | cy**MPO**  Clone: MPO-7  Dako | **CD34**  Clone:8G12  BD Biosciences | **CD13**  Clone: Immu103.44  Beckman Coulter | **CD117**  Clone: YB5.B8  BD Pharmingen | **HLA-DR**  Clone:G46-6  BD Biosciences | **CD11b**  Clone: ICRF44  BD Pharmingen | **CD45**  Clone: J.33  Beckman Coulter |


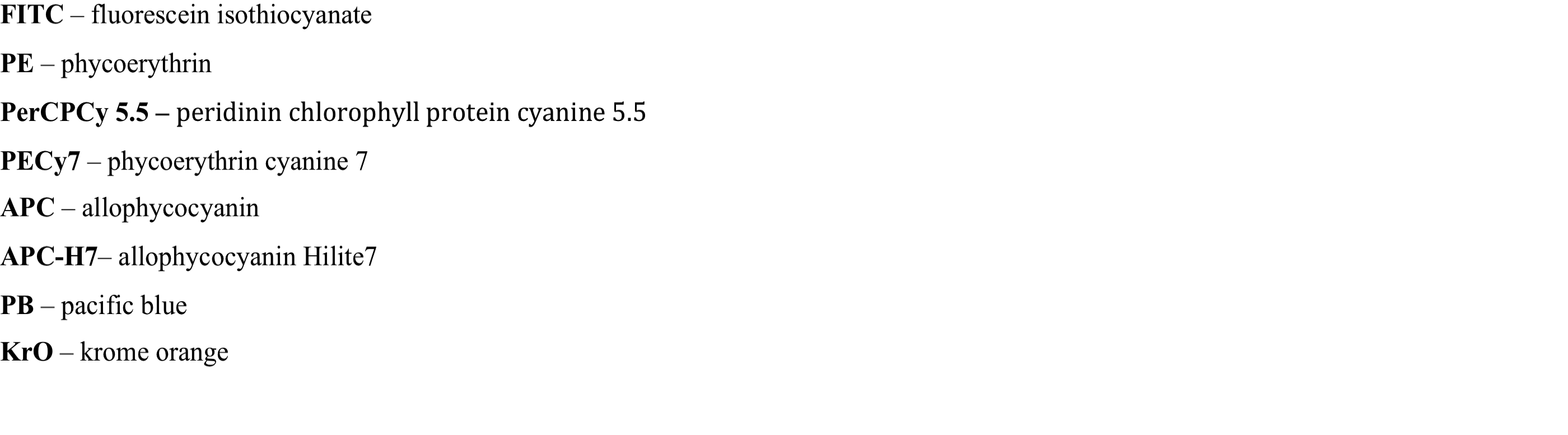


| **Immature (uncommitted) CD34^+^ cells:**  CD34^hi^/CD45^hi^/HLA-DR^int^/CD117^int^/CD133^hi^/FSC^int^/SSC^int^/CD123^-^/cyMPO^-^/CD13^int^/CD11b^-^ |
| --- |
| **Neutrophil lineage**:  CD34^+int^/CD133^int^/CD35^-^/HLA-DR^hi^/CD117^hi^/CD123^-^/CD45^int/dim^/FSC^hi^/SSC^hi^/cyMPO^+^/CD13^hi^/CD11b/CD44^hi^ |
| **B-cell lineage**:  CD34^dim^/CD45^int/dim^/HLA-DR^hi^/CD123^-^/CD117^-^/CD44^dim^/FSC^low^/SSC^low^/cyMPO^-^/CD13^-^ |
| **Monocytic lineage**:  CD34^int/dim^/HLA-DR^hi^/CD117^low/-^/CD44^dim/-^/CD64^hi^/CD45^hi^ |
| **Plasmacytoid dendritic cells lineage**:  CD34^+^/HLA-DR^hi^/CD123^hi/int^/CD117^+dim/-^/CD44^high^ |
| **Erythroid lineage:**  CD34^+^/HLA-DR^int/low^/CD45^low^/CD123^-^/CD117^+/int^/CD44^dim^/CD71^+^/CD36^+^/CD13^+dim/-^/cyMPO^-^/ |
| **Basophil lineage**:  CD34^+^/HLA-DR^+dim/-^/CD123^int/hi^/CD117^+dim/-^/CD44^low^/ |
| **Mast Cells lineage:**  CD34^+^/HLA-DR^+int/dim^/CD117^+/int/high^/CD44^hi^ |
